# The Effectiveness of Dance Interventions on Psychological and Cognitive Health Outcomes Compared with Other Forms of Physical Activity: A Systematic Review with Meta-analysis

**DOI:** 10.1007/s40279-023-01990-2

**Published:** 2024-01-25

**Authors:** Alycia Fong Yan, Leslie L. Nicholson, Rachel E. Ward, Claire E. Hiller, Kathryn Dovey, Helen M. Parker, Lee-Fay Low, Gene Moyle, Cliffton Chan

**Affiliations:** 1 Sydney School of Health Sciences, Faculty of Medicine and Health, The University of Sydney, Sydney, NSW Australia; 2 School of Medical Sciences, Faculty of Medicine and Health, The University of Sydney, Sydney, NSW Australia; 3 School of Health Sciences, Faculty of Medicine and Health, UNSW Sydney, Sydney, NSW Australia; 4 Faculty of Creative Industries, Education and Social Justice, Queensland University of Technology, Brisbane, QLD Australia; 5 Department of Health Sciences, Faculty of Medicine, Health and Human Sciences, Macquarie University, Sydney, NSW Australia

## Abstract

**Background:**

Physical activity is known to improve psychological and cognitive outcomes. Learning dance sequences may challenge cognition, partnered or group dance may benefit social interactions, and the artistic aspect may improve psychological wellbeing. Dance is an equally effective form of physical activity compared with other structured physical activities to improve physical health, but it is unclear how effective dance could be for psychological and cognitive outcome measures.

**Objective:**

To systematically review the literature on the effectiveness of structured dance interventions, compared with structured exercise programmes, on psychological and cognitive outcomes across the lifespan.

**Methods:**

Eight databases were searched from earliest records to July 2022. Studies investigating a dance intervention lasting ≥ 4 weeks, including psychological and/or cognitive health outcomes, and having a structured exercise comparison group were included. Screening and data extraction were performed by two independent reviewers at all stages. All reviewer disagreements were resolved by the primary author. Where appropriate, meta-analysis was performed, or an effect size estimate generated.

**Results:**

Of 21,737 records identified, 27 studies met the inclusion criteria. Total sample size of included studies was 1392 (944 females, 418 males, 30 unreported). Dance was equally as effective as other physical activity interventions in improving quality of life for people with Parkinson’s disease [mean difference 3.09; 95% confidence interval (CI) − 2.13 to 8.30; *p* = 0.25], reducing anxiety (standardised mean difference 2.26; 95% CI − 2.37 to 6.90; *p* = 0.34), and improving depressive symptoms (standardised mean difference 0.78; 95% CI − 0.92 to 2.48; *p* = 0.37). Preliminary evidence found dance to be superior to other physical activity interventions to improve motivation, aspects of memory, and social cognition and to reduce distress. Preliminary evidence found dance to be inferior to other physical activity interventions to improve stress, self-efficacy and language fluency.

**Conclusion:**

Undertaking structured dance of any genre is generally equally and occasionally more effective than other types of structured exercise for improving a range of psychological and cognitive outcomes.

**Trial Registration:**

PROSPERO: CRD42018099637.

## Key Points

**Table:** 

Structured dance of at least 6 weeks’ duration can significantly improve psychological and cognitive health outcomes equivalent to other forms of structured exercise or physical activity.
Preliminary evidence suggests dance may be better than other physical activities to improve emotional wellbeing, depression, motivation, social cognition and some aspects of memory.

## Introduction

### Physical Activity Effect on Psychological and Cognitive Health

The lifetime prevalence of mental disorders is estimated to be between 26 and 33%, accounting for 9% to nearly 17% of the total burden of all disease worldwide [1, 2]. Those affected have 10–15 years shorter life expectancy, up to three times higher risk of developing a chronic disease, and lower quality of life [3–5]. The most common mental health problems are depression and anxiety, contributing to the leading causes of death worldwide (e.g. death by suicide, coronary heart disease and stroke) [6–8]. While mental illness can affect people across the lifespan, cognitive impairment refers to cognitive decline greater than expected with normal ageing. Cognitive impairment is linked to poorer medication adherence, poor physical and mental health, and higher morbidity and mortality associated with increased cost of care and caregiver burden [9–11].

Exercise is a cost-effective treatment shown to significantly improve psychological health and cognitive functioning as well as positively alter health beliefs, ultimately lowering the healthcare burden driven by mental illness [12–16]. Some mechanisms by which exercise achieves these outcomes are through triggering an increase in endorphin levels, neurotransmitter production and distraction from feelings of depression and anxiety [17–19]. Despite the proven efficacy of exercise, 25% of adults are physically inactive [20]. Further, 50% of people who start an exercise programme will cease within half a year, while the adherence rates in community-based and clinical trials are at best moderate at 52% and 66%, respectively [21, 22]. Therefore, the provision of various exercise options that facilitate higher rates of adherence is warranted.

### Dance as a Physical Activity Modality

Dance is a unique form of physical activity requiring complex movements combined with aesthetics, music, choreographed movement sequences and planned interactions with other people. A previous systematic review and meta-analysis of the literature reported that structured weekly dance sessions over 6 or more weeks improved a number of physical health measures to the same extent (e.g. cardiovascular function) or were superior (e.g. body mass index reduction) to other types of structured exercise [23].

Meeting the physical activity recommendations and adherence to physical activity is the biggest challenge for people to achieve improvements to health [24]. Dance can be performed without much equipment and is associated with higher clinical trial completion rates of 86–100% [25, 26]. Although there is limited substantiated evidence, it has been proposed that enjoyment of dance may increase adherence to physical activity [27], thereby enhancing health benefits.

Several elements may be responsible for the potential mental health effects of dance as a form of exercise. Exercise in general has been found to improve cognition in both healthy populations [28], as well as populations with cognitive conditions such as attentional deficit hyperactivity disorder [29] and mild cognitive impairment, who may indeed be able to expect greater positive cognitive outcomes from participating in physical exercise [30]. A recent systematic review found that, while aerobic endurance exercise produces improvements in general and domain-specific cognition, coordinative exercise resulted in larger effect sizes for improvement in cognitive function than endurance, resistance, or mixed exercise [28]. In addition, exercising in a group setting has been shown to have additional mental health benefits as compared with exercising alone [31]. Dance, which is often undertaken in a group setting, requires aerobic endurance and is an inherently coordinative activity, incorporating elements of learning and mastering individual steps, performing those steps in a set order, and then remembering those sequences while performing them in time to music. Dance provides participants with additional health benefits due to the social and interactive aspects, its enjoyable nature and its unique cognitive challenges [32].

### Dance and Psychological Health

The supportive social nature and partnership of dance enhances participant enjoyment [33] and provides social engagement, accountability and psychological relaxation which may assist with managing mental disorders, such as anxiety and depression [26, 33, 34]. Socialising both in and after dance class generates a sense of community and kinship that adds to the dancer's self-identity and mental wellbeing [35].

Dance participation often requires dancers to touch, make eye contact and synchronise movements to music, an instructor, a partner or a group. Performing exertive movements in synchrony with others has been linked with release of endorphins [36, 37], a peptide implicated in the social bonding often experienced during group musical activities [38]. Additionally, performing movements simultaneously with others is suggested to cause some ‘merging of self and other’ via the neural pathways coding for action and perception [39]. Other properties of dance participation that can increase social bonding, and therefore psychological health, relate to shared intention and cooperation [40] and increased physical awareness of self in relation to others in space [41]. The experience of trustful interpersonal touch, which often occurs during participation in dance, has been associated with beneficial effects such as enhanced homeostatic regulation and immunoregulation [42] and increased body appreciation [41]. The process of dancers focusing on their body from within during the ‘here and now’ has been proposed to induce mindfulness, a state of present-centred awareness [42] and is reported to promote improved coping skills and self-regulation [43].

In a systematic review and meta-analysis investigating the effect of dance on cognition, depression and quality of life in older adults, the majority of the included studies utilised non-active comparator groups such as usual care, wait list and/or education [44]. Dance for Parkinson’s disease was shown to be successful at alleviating symptoms of depression and anxiety and improving quality of life and some cognitive skills in one study; however, the comparator group was non-active [45]. Whilst many forms of exercise are known to have favourable effects on psychological and cognitive health compared with non-active comparators, the gap in knowledge now lies in relation to the effectiveness of dance relative to an active comparator.

### Dance and Cognition

Dancing requires superior motor planning and memory, multitasking, and focused and conscious attention [45] to learn new movement patterns, remember choreographed sequences of movement [44, 46, 47], moving in time with the music, and particular focus and attention on movement quality and artistic expression. Imaging studies have suggested that dance has neuroprotective effects, preventing age-related degeneration of the brain for memory functions and increasing resting-state activity in the fronto-temporal areas assumed to maintain memory and cognitive functions [48, 49].

Current studies investigating the effects of dance on cognition highlight the need to control for various aspects of intervention design. Tango dancing improved motor–cognitive performance in a body position spatial task and maintained cognitive function of older adults. However, as the comparator group was not active, it is unknown if a standard exercise programme would produce similar results [50]. Kattenstroth and colleagues [51] reported that older adults, with a history of participating in regular recreational dance, performed better on two different cognitive tests compared with the control group of non-dancers or participants in sporting activities. Similarly, older adults with metabolic syndrome showed improved cognition in verbal fluency and memory function when compared with a non-active control group [52]. Given the evidence supporting the role of structured exercise to improve cognition, a truly active comparator group would further our understanding of the effectiveness of dance as an intervention.

The value of physical activity combined with care and instruction from a clinician or movement instructor should not be overlooked when assessing the efficacy of dance. Kosmat and Vranic [53] accounted for the potential confounding effect of therapist contribution by ensuring the active control group met with a researcher for the same time and frequency as the dance group so that any effect of therapeutic alliance [54] would have the same dosage [55]. When a comparator group has the potential to gain similar health benefits from being physically active and may have the additional improvements due to personal care from a clinician or movement instructor, then it will be easier to determine the potency of dance as an alternative modality.

### Study Purpose

The aim of this study was to systematically review the literature to investigate the effectiveness of structured dance interventions on psychological and cognitive outcomes compared with other forms of structured physical activity across the lifespan. We hypothesised that dance may improve psychological and cognitive health outcomes that are influenced by social engagement and support. The secondary aim of this systematic review was to compare the rates of adherence between structured dance interventions and structured physical activity programmes. Our null hypothesis was that adherence to dance would be equal to adherence to structured physical activity programmes. Understanding how dance can impact psychological and cognitive health can assist clinicians to recommend dance for specific psychological and cognitive health outcomes.

## Methods

The protocol for this review was registered in the PROSPERO international prospective register of systematic reviews (CRD42018099637). The process of completing and reporting this review adhered to the Preferred Reporting Items for Systematic Reviews and Meta-analysis (PRISMA) Statement guidelines [56].

### Inclusion Criteria

Studies were included if they were a full original peer-reviewed study that compared a structured dance intervention with any other form of physical activity. Dance classes were required to have an instructor teaching any genre of dance with choreographed movement sequences, and planned interactions with other people. Dance classes needed to occur for at least 4 weeks to ensure potential psychological or cognitive changes would be evident. Outcome measures had to include a psychological or cognitive health outcome(s) through scales, examinations or surveys for example. There were no restrictions imposed on age, clinical condition or language.

### Exclusion Criteria

Excluded were systematic reviews, narrative reviews or individual case studies; those that reported only on physical health outcomes; those where dance groups were combined with other forms of exercise or physical activity; those where dance movement therapy or improvised dance was examined; or studies where the control group did not include physical activity.

### Systematic Search

Eight electronic databases were searched: MEDLINE, Embase, PEDro, Cochrane Central Register of Controlled Trials (CENTRAL), SPORTDiscus, Web of Science, Scopus and PsycINFO. No language or publication date restrictions were imposed, and the last search was completed in July 2022. The authorship team developed the search strategy, with the assistance of an experienced medical librarian. The search terms used to identify the articles were: [danc* or ballet or ballroom or jazz or ballroom or salsa or tango or folk] AND [depress* or mood* or memory or anxi* or “social engagement” or “quality of life” or fear* or stress or empowerment or “response inhibition” or well-being or cognit* or “executive function” or “information processing” or self-efficacy or self-esteem or “mental health” or wellness]. MESH headings were searched for additional key words to be included.

After conducting the search, one of the authors (K.D.) removed all duplicates. Screening of titles and abstracts of the remaining records was completed by two independent reviewers of the authorship team. Full-text articles were then retrieved for all remaining records and screened by two independent reviewers of the authorship team. In the case of uncertainty as to whether a study met inclusion criteria, cases were resolved by the primary author (A.F.Y.). Reference lists of each included study were examined to identify and locate potential additional studies.

### Data Extraction

From identified studies, specific information and data were then extracted by two independent reviewers of the authorship team. Information included author, year, participant characteristics, population studied, details of the dance intervention, details of the active comparator, session duration, frequency, study length and follow-up, retention (calculated as the proportion of the initial sample size that completed the study) and adherence to the intervention (calculated as the proportion of sessions participated in of the total sessions offered). Missing information was denoted as ‘not reported’. The accuracy and veracity of extracted data was checked by two reviewers (L.N. and C.C.).

### Study Quality Assessment

Two independent reviewers of the authorship team assessed the study quality of each included study. Two different critical appraisal tools were used as appropriate to the study design. The first was a 13-item tool for assessing risk of bias in randomised controlled trials [57], and the second was a 9-item tool for assessing risk of bias in non-experimental studies [57]. There was no exclusion of studies based on the quality assessment results. Disagreements were resolved by discussion between assessors.

### Quantitative Analysis

During data extraction, there were several studies which examined similar psychological outcomes, using the same tool. Therefore, with complete pre- and post-intervention data reporting, a meta-analytical synthesis became feasible. When two or more studies examined a given outcome variable (e.g. PDQ-39), raw mean and standard deviation data at pre- and post-intervention stages for both dance and comparison exercise groups were transferred to Review Manager software (RevMan [PC] Version 5.4, the Cochrane Collaboration, 2020). Data related to two psychological outcomes were transferred for independent analysis. For each, an invariance random effects model was applied [58], assuming studies drew potentially from divergent populations and contexts; included possibly different research designs; and tested different dance genres and had different exercise comparison groups. Thus, a true exact effect size was not expected to exist across studies. Unadjusted mean differences (MD) and standardised mean differences (SMD) were applied depending on whether the psychological outcome variables used consistent forms of measurement. Overall pooled estimates and confidence intervals indicated the effect of dance versus exercise comparison interventions and were summarised in forest plots. The *Z* and *p* values tested the null hypothesis that pooled estimates of dance groups were no more effective than exercise comparison groups. As MD analyses retained their common unit metric of measurement, their functional meaning and interpretation were retained. Meanwhile, as SMD is on a standard deviation scale and does not have a unit of measurement, effect sizes were classified as small (± 0.2) moderate (± 0.5) and large (± 0.8) on the basis of Cohen’s recommendations [59]. The *I*^2^ statistic identified the proportion of observed variance reflecting differences in true effect sizes as opposed to sampling error. If found, moderate (≥ 0.50) to high values (> 75%) were used to suggest heterogeneity in effect sizes between studies. Where studies reported that dance had a greater significant effect compared with the standard exercise comparator and did not fulfil criteria for meta-analysis but still reported complete data (i.e. pre–post dance and exercise means, standard deviations and sample size), study effect size estimates were generated using the Hedges’ *g* statistic [60]. Again, effect sizes were classified as small (± 0.2) moderate (± 0.5) and large (± 0.8), on the basis of the positive or negative direction of the favoured trend [61].

## Results

### Study Identified and Characteristics

**Fig. 1:**
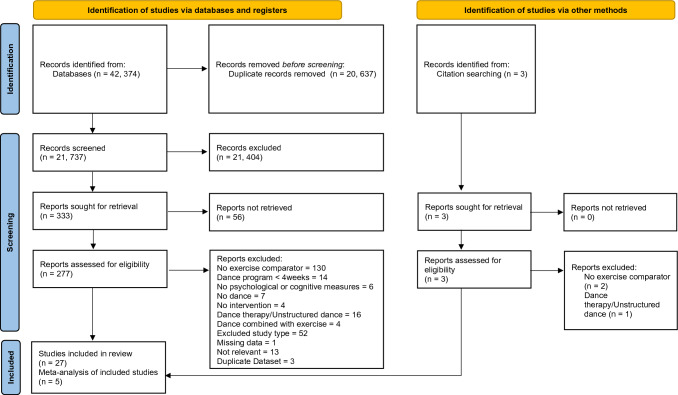
PRISMA [56] flowchart of studies through the selection process

A total of 42,374 articles were found. After duplicates were removed there were 21,737 articles which were screened by title and abstract and 21,404 were excluded (Fig. 1). Following full text review of the remaining 277 retrievable and three hand-searched articles, 27 studies were included in the review. Figure 1 summarises the systematic search and study identification process, including reasons for exclusion.

### Study Quality

Tables 1 and 2 summarise study quality assessments. All studies were of low to moderate quality. Nineteen studies were randomised controlled trials (RCTs) [62–75], and eight were non-RCTs [76–83]. Of the 19 RCTs, only 2 studies clearly stated that participants were blinded [64, 66]. All but two studies [70, 73] clearly used appropriate statistical analysis and study design. The non-RCTs all measured outcomes in a reliable way, and all but one [81] clearly described appropriate statistical analysis. However, description of the participants in each group, description of whether the groups were receiving equivalent treatments outside the intervention, and reporting of follow-up were largely unclear or missing. Neville and Makopoulou (2021) was the only study to receive ‘yes’ responses to all items of the critical appraisal tool [82].

**Table 1:** Critical appraisal of the included studies using the Johanna Briggs Institute Critical Appraisal Tool for RCTs [57]

	Barene and Krustrup 2022 [62]	Bisbe et al. 2020 [63]	Gray et al. 2018 [84]	Hamacher et al. 2015 [64]	Kaltsatou et al. 2014 [65]	Lee et al. 2009 [66]	Leite et al. 2021 [85]	McKinley et al. 2008 [67]	Merom et al. 2016 [68]	Müller et al. 2017 [69]	Norouzi et al. 2020 [86]	Nørregaard et al. 1997 [70]	Oppici et al. 2020 [71]	Poier et al. 2019 [72]	Rodziewicz-Fils et al. 2022 [88]	Salamuddin et al. 2014 [73]	Teixeira-Machado et al. 2017 [74]	Volpe et al. 2013 [75]	Voss et al. 2019 [89]
True randomisation used for assignment of participants to treatment groups?	?	✔	✔	✔	✔	✔	✔	✔	✔	✔	?	✔	×	✔	✔	✔	✔	✔	✔
Was allocation to treatment groups concealed?	?	✔	✔	✔	✔	?	?	✔	✔	✔	?	✔	?	?	?	?	✔	✔	?
Were treatment groups similar at the baseline?	✔	×	✔	✔	✔	×	?	✔	×	✔	✔	×	✔	✔	✔	?	✔	✔	✔
Were participants blind to treatment assignment?	×	×	×	✔	?	✔	×	×	?	×	×	×	×	×	×	×	×	?	×
Were those delivering treatment blind to treatment assignment?	×	×	×	×	×	×	×	×	?	×	×	×	×	×	×	×	×	?	×
Were outcomes assessors blind to treatment assignment?	?	✔	?	✔	✔	?	✔	✔	×	?	?	✔	✔	?	?	?	?	✔	?
Were treatment groups treated identically other than the intervention of interest?	✔	✔	✔	✔	✔	✔	✔	✔	✔	✔	✔	×	✔	✔	?	✔	✔	?	✔
Was follow-up complete? If not, were follow-up differences between groups adequately described and analysed?	×	✔	?	✔	✔	✔	✔	✔	✔	✔	✔	✔	✔	✔	✔	?	✔	?	✔
Were participants analysed in the groups to which they were randomised?	✔	×	✔	×	×	✔	✔	✔	✔	✔	✔	×	✔	✔	✔	✔	✔	?	✔
Were outcomes measured in the same way for treatment groups?	✔	✔	✔	✔	✔	✔	✔	✔	✔	✔	✔	✔	✔	✔	✔	✔	✔	✔	✔
Were outcomes measured in a reliable way?	✔	✔	✔	✔	✔	✔	✔	✔	✔	✔	✔	?	✔	✔	✔	✔	✔	✔	✔
Was appropriate statistical analysis used?	✔	✔	✔	✔	✔	✔	✔	✔	✔	✔	✔	?	✔	✔	✔	?	✔	✔	✔
Was trial design appropriate, and any deviations from standard RCT design accounted for in conduct and analysis?	?	✔	✔	✔	✔	✔	✔	✔	✔	✔	✔	?	✔	✔	✔	?	✔	✔	✔

✔ Yes, × No, ? Unclear

**Table 2:** Critical appraisal of the included studies using the Johanna Briggs Institute Critical Appraisal Tool for Quasi-experimental Studies [57]

	Bass et al. 2002 [76]	Chuang et al. 2015 [77]	Dahmen-Zimmer and Jansen 2017 [78]	Johnston et al. 2021 [81]	Leste and Ruste 1984 [79]	Neville and Makopoulou 2021 [82]	Rawson et al. 2019 [83]	Soares Costa de Mendonca et al. 2015 [80]
Is it clear in the study what is the ‘cause’ and what is the ‘effect’ (i.e. there is no confusion about which variable comes first)?	✔	✔	✔	?	✔	✔	✔	✔
Were the participants included in any comparisons similar?	×	?	?	?	×	✔	✔	?
Were the participants included in any comparisons receiving similar treatment/care, other than the exposure or intervention of interest?	?	?	?	✔	?	✔	✔	?
Was there a control group?	✔	✔	✔	✔	✔	✔	✔	✔
Were there multiple measurements of the outcome both pre and post the intervention/exposure?	✔	✔	✔	✔	✔	✔	✔	✔
Was follow-up complete, and, if not, were differences between groups in terms of their follow-up adequately described and analysed?	?	?	?	?	?	✔	?	?
Were the outcomes of participants included in any comparisons measured in the same way?	✔	✔	✔	✔	✔	✔	✔	✔
Were outcomes measured in a reliable way?	✔	✔	✔	✔	✔	✔	✔	✔
Was appropriate statistical analysis used?	✔	✔	✔	×	✔	✔	✔	✔

✔ Yes, × No, ? Unclear

### Summary of Studies

Studies included between 18 and 291 participants, with a combined total of 1392 participants (944 females, 418 males, 30 unreported). Nine population groups were represented: healthy people (*n* = 14), those with Parkinson’s disease (*n* = 4), low cognition (*n* = 3), cerebral palsy (*n* = 1), fibromyalgia (*n* = 1), generalised musculoskeletal pain (*n* = 1), unexplained falls (*n* = 1), breast cancer (*n* = 1) and chronic heart failure (*n* = 1). The included studies were categorised into age groups (Table 3).

**Table 3:** Demographic data for the included studies

Study	Total sample size	Allocation sample size	Sex F:M	Age range (mean ± standard deviation)	Population
Barene and Krustrup 2022 [62]	69	I: 34 C: 35	69:0	25–65 I: 46.3 ± 9.5 C: 44.8 ± 8.9	Hospital employees
Bass et al. 2002 [76]	80	I: 35 C: 45	48:32	17–29 (NR)	University students
Bisbe et al. 2020 [63]	31	I: 17 C: 14	15:16	65–85 I: 72.9 ± 5.6 C: 72.3 ± 5.2	Older adults with amnestic mild cognitive impairment
Chuang et al. 2015 [77]	18	I: 7 C: 11	18:0	65–75 I: 69.4 ± 3.8 C: 76.0 ± 1.7	Sedentary elderly females
Dahmen-Zimmer and Jansen 2017 [78]	25	I: 12 C: 25	10:27	NR I: 73.4 ± 6.7 C: 68.9 ± 7.2	Parkinson’s disease
Gray et al. 2018 [84]	23	I: 11 C: 12	23:0	NR I: 63.8 ± 4.3 C: 61.8 ± 5.5	Community-dwelling women not meeting MVPA
Hamacher et al. 2015 [64]	35	I: 24 C: 25	21:14	NR I: 67.2 ± 3.4 C: 68.5 ± 3.1	Sedentary older healthy adults MMSE less than 27/30
Johnston et al. 2021 [81]	291	I: 153 C: 138	220:71	NR I: 18.3 ± 1.2 C: 18.9 ± 1.3	University students
Kaltsatou et al. 2014 [65]	38	I: 19 C: 19	0:38	NR I: 67.2 ± 4.2 C: 67.1 ± 7.2	Stage 2 and 3 heart failure
Lee et al. 2009 [66]	48	I: 21 C: 27	48:0	NR (Whole cohort 13.3 ± 0.1)	Female youth
Leite et al. 2021 [85]	36	I: 18 C: 18	36:0	NR I: 53.0 ± 10.0 C: 53.0 ± 8.0	Patients with breast cancer (stages 0–3) during hormonal therapy
Leste and Rust 1984 [79]	50	I: 29 C: 21	39:11	NR [Whole cohort 19.9 (no SD)]	Tertiary students
Mendonça et al. 2015 [87]	64	I: 18 C1: 25 C2: 21	64:0	22–55 (Whole cohort 41.4 ± 9.2)	Healthy females
Merom et al. 2016 [68]	115	I: 61 C: 54	88:27	NR (Whole cohort 69.5 ± 6.4)	Healthy, older adults
McKinley et al. 2008 [67]	25	I: 14 C: 11	19:6	NR I: 78.1 ± 7.6 C: 74.6 ± 8.4	60+ years, 1+ unexplained fall in previous year
Müller et al. 2017 [69]	22	T1 I: 20 C: 18 T2 I: 12 C: 10	10:11	NR I: 68.3 ± 3.9 C: 68.6 ± 2.8	Healthy, older adults
Neville and Makopoulou 2021 [82]	40	I: 20 C: 20	20:20	I: 7–8 (7.4 ± 0.5) C: 7–8 (7.4 ± 0.5)	Primary school children
Norouzi et al. 2020 [86]	40	I: 20 C: 20	40:0	I: 30–40 (35.5 ± 2.4) C: 30–40 (35.5 ± 2.4)	Mid-aged females with fibromyalgia
Nørregaard et al. 1997 [70]	30	I: 5 C: 11	NR	NR I: 44.0 ± 8.0 C: 51.0 ± 1.0*	Generalised musculoskeletal pain
Oppici et al. 2020 [71]	74	I1: 26 I2: 29 C: 19	50:30	I1: 8–10 (8.8 ± 0.5) I2: 8–10 (8.7 ± 0.7) C: 8–10 (8.9 ± 0.7)	Primary school children with high or low cognition
Poier et al. 2019 [72]	29	I: 14 C: 15	17:12	NR I: 68.5 ± 8.1 C: 68.9 ± 11.0	People with Parkinson's disease aged 50–90 years
Rawson et al. 2019 [83]	70	I: 39 C: 31	28:42	NR I: 66.7 ± 9.5 C: 68.5 ± 9.5	People with Parkinson's disease aged over 30 years
Rodziewicz-Flis et al. 2022 [88]	20	I: 10 C: 10	20:0	65–82 I: 72.1 ± 4.1 C: 74.3 ± 4.6	Community-dwelling older women
Salamuddin et al. 2014 [73]	60	I: 30? C: 30?	60:0	NR	Undergraduate sedentary females
Teixeira–Machado et al. 2017 [74]	26	I: 13 C: 13	15:11	NR I: 17.1 ± 2.4 C: 18.0 ± 3.5	Cerebral palsy
Volpe et al. 2013 [75]	24	I: 12 C: 12	11:13	56–73 I: 61.6 ± 4.5 C: 65.0 ± 5.3	Parkinson’s disease
Voss et al. 2019 [89]	124	I: 46 C1: 43 C2: 35	87:37	NR I: 65.7 ± 4.6 C1: 65.9 ± 4.3 C2: 65.5 ± 4.7	Community-dwelling older adults aged 60–80

*F* female, *M* male, *NR* not reported, *I* intervention group, *C* comparator group, *T1* first follow-up, *T2* second follow-up, *?* assumed, *MVPA* moderate to vigorous physical activity, *MMSE* Mini-Mental State Examination

*Significant difference reported between groups

Descriptions of interventions used in the included studies are presented in Table 4. Dance interventions consisted of both partnered and individual, with the following dance genres included: aerobic dance (Zumba, step dance), theatrical dance styles (modern, jazz), ballroom (rumba, waltz, jive, Latin American, salsa, rock ‘n roll), dance gaming, dance-based physical education, and cultural dances (line dance, square dance, qi gong, belly dance, folk dances of Brazil, Poland, England, Ireland, Greece and Argentina). The dance sessions varied in duration from 30 min [77] to 90 min [64, 67, 69, 75, 81] per session. Frequency of sessions ranged from once per week [72, 75, 78, 81, 82] to four times per week [73]. One study [79] did not report on session length or frequency. Follow-up ranged from 6 weeks [82, 84] to 18 months [69].

**Table 4:** Intervention details of included studies and summary of findings

Study	Intervention	Comparator	Session duration (min)	Session frequency (per week)	Study length	Retention	Outcome measure(s)	In favour of dance
Barene and Krustrup 2022 [62]	Zumba	Soccer	60	T1: 3 T2: 2	40 weeks T1: 12 weeks T2: 28 weeks	T1 I: 85% C: 83% T2 I: 59% C: 49%	Psychological Self-efficacy—3 items from 10-item Generalized Self Efficacy scale Emotional wellbeing—5 items from RAND 36 Item Health Survey	T1: N T2: = T1: N T2: =
Bass et al. 2002 [76]	Aerobic dance	Weight training	45^a^	3	8 weeks	NR	Psychological Stress—Survey of Recent Life Experiences	N*
Bisbe et al. 2020 [63]	Choreographed dances (Salsa, rock, rumba, pop, jive)	Physical therapy (Strength, endurance, flexibility, gait, balance)	60	2	12 weeks	I: 94% C: 78%	Cognitive Verbal memory—WMS-III Delayed recall Recognition Visual memory—RBANS Delayed recall Recognition Processing speed—Trail Making Test -A Executive function—Trail Making Test -B Executive function—Letter Verbal Fluency Language—Boston Naming Test Language—Category Verbal Fluency Visuospatial—Judgment Line Orientation Global cognition—MMSE Psychological Anxiety—HADS Depression—HADS QoL—Short Form 36	= Y = = = = = = N = = = = =
Chuang et al. 2015 [77]	Dance Dance Revolution	Brisk walking	30	3	12 weeks	I: 88% C: 85%	Cognitive Executive function—Flanker Task	=
Dahmen-Zimmer and Jansen 2017 [78]	Line dance, rumba and waltz movements	Karate (Kihon, Kumite and Kata)	60	1	30 weeks	I: 75% C: 64%	Cognitive Cognitive processing—Number Connection Test General cognitive ability—PANDA Psychological Anxiety and depression—HADS Emotional wellbeing—MDBF QoL—12-item Short Form Health Survey Self-efficacy—Short Scale of General Self-Efficacy (not reported in Results)	* = = = = = N?
Gray et al. 2018 [84]	Dance (genre unspecified)	Walking	60	2	6 weeks	I: 97% C: 100%	Psychological Psychological Need Satisfaction in Exercise Scale (PNSE)	=
Hamacher et al. 2015 [64]	Jazz, Latin American, rock’n’roll, line and square dance	Health-related exercise	90	2	6 months	I: 79% C: 64%	Cognitive Dual task—Successive Subtraction	=
Johnston et al. 2021 [81]	Aerobic dance	Team sport (Volleyball or soccer)	90	1	12 weeks	I: 100% C: 100%	Psychological Anxiety—Generalized Anxiety Disorder Scale (GAD-7) Stress—Perceived Stress Scale (PSS) Depression—Center for Epidemiologic Studies Depression Scale (CESD-R)	= = N
Kaltsatou et al. 2014 [65]	Greek dance	Formal exercise	50–60	3	8 months	I: 95% C: 84%	Psychological QoL—Short Form 36 QoL—Life Satisfaction Inventory Motivation—Intrinsic Motivation Inventory Enjoyment/interest Effort/importance Perceived competence Pressure/tension	= = Y Y Y Y =
Lee et al. 2009 [66]	Qi gong dancing	Similar movements, without the concept of meridian Qi flow	40	2	2 months	I: 100% C: 100%	Cognitive Mental stress—Stroop or Mental Arithmetic Psychological Distress—Symptom Checklist 90 Revision Hostility Somatisation Obsession Compulsion Interpersonal Sensitivity Depression Anxiety Phobic Anxiety Paranoid Ideation Psychoticism	= = Y Y = = = = = = =
Leite et al. 2021 [85]	Belly dance	Mat Pilates (Stotts)	60	3	16 weeks	I: 72% C: 72%	Psychological Depression—Beck Depression Inventory (BDI) Self-esteem—Rosenberg Self Esteem Scale	= =
Leste and Rust 1984 [79]	Modern dance	Physical education	NR	NR	3 months?	I: 79% C: 76%	Psychological Anxiety—Spielberg State & Trait Anxiety Inventory	= *
Mendonça et al. 2015 [87]	Brazilian folk dance	Strength hydrogymnastic	50–60	3	16 weeks	NR	Psychological Self-esteem—Rosenberg Self Esteem Scale Body image perception—Scale of Stunkard silhouettes Satisfaction with physical appearance—Likert scale Perception of health—Likert scale Depression—Beck Depression Inventory	= = = = =
Merom et al. 2016 [68]	Ballroom	Walking	60	2	8 months	I: 66% C: 72%	Cognitive Executive function—Trail Making Tests Visuospatial immediate and delayed memory—Brief Visuospatial memory test Response inhibition—Stroop Working memory—Digits Span Backwards Verbal immediate and delayed memory and learning—Rey Auditory verbal learning test Verbal intelligence—Spot the Word Test	= = = = = =
McKinley et al. 2008 [67]	Argentine tango	Walking	90	2	10 weeks	I: 93% C: 64%	Cognitive Cognitive function—Folstein Mini-Mental Status Test Psychological Balance confidence—ABC	= =
Müller et al. 2017 [69]	Line, jazz, rock’n’roll, square dance	Strength and endurance training	90	T1: 2 T2: 1	18 months T1: 6 months T2: 12 months	T1 I: 77% C: 69% T2 I: 60% C: 56%	Cognitive Attention—Test of Attentional Performance Verbal immediate and delayed memory and learning—Rey Auditory verbal learning test	= (T1 &T2) = (T1 &T2)
Neville and Makopoulou 2021 [82]	Dance-based physical education	Sport-based physical education	50	1	6 weeks	I: 100% C: 100%	Cognitive Creativity—Alternative Uses Test	=
Norouzi et al. 2021 [86]	Zumba	Aerobic exercise (treadmill)	60	3	12 weeks	I: 100% C: 100%	Cognitive Working memory—N-Back Task Psychological Depression—Beck Depression Inventory (BDI-II)	Y Y
Nørregaard et al. 1997 [70]	Aerobic dance	Steady-state exercise	50	3	12 weeks	I: 33% C: 73%	Psychological Depression—Beck Depression Inventory (BDI)	=
Oppici et al. 2020 [71]	Jazz dance	Sport-based physical education	55	2	7 weeks	I: 75% (high cog group); 97% (low cog group) C: 95%	Cognitive Working memory—National Institute for Health Toolbox Flexibility—Dimensional Change Card Sort Test Executive function—Flanker Test	= = =
Poier et al. 2019 [72]	Argentine Tango	Tai Chi	60	1	10 weeks	I: 78% C: 79%	Cognitive General cognition—PDQ-39 (sub score) Psychological Emotional wellbeing—PDQ-39 (sub score) Stigma—PDQ-39 (sub score) Social support—PDQ-39 (sub score) Life satisfaction—Brief Multidimensional Life Satisfaction Scale Inner congruence—Inner Correspondence and Feelings of Peaceful Relief	= Y = = = =
Rawson et al. 2019 [83]	Argentine Tango	Treadmill	60	2	12 weeks	I: 91% C: 76%	Psychological QoL—PDQ39 (total score)	=
Rodziewicz-Flis et al. 2022 [88]	Polish Folk Dance	Balance training	50	3	12 weeks	I: 70% C: 80%	Psychological Reactive stress tolerance—Determination Test (Vienna Test System)	=
Salamuddin et al. 2014 [73]	Step-dance aerobics	Walking and jogging	60	4	12 weeks	NR	Psychological Self-esteem—Rosenberg Self Esteem Scale	= ?**
Teixeira- Machado et al. 2017 [74]	Modern dance based on somatic methods	Kinesiotherapy	60	2	3 months	I: 100% C: 100%	Cognitive Social cognition—Functional Independence Measure Psychosocial adjustments Cognitive function	Y Y
Volpe et al. 2013 [75]	Irish dancing	Physio exercises	90	1	6 months	I: 100%? C: 100%?	Psychological QoL—PDQ39 (total score)	=
Voss et al. 2019 [89]	Contra and English Country Dancing	C1: strength, stretch and stability training C2: walk	60	3	24 weeks	I: 87% C1: 83% C2: 90%	Cognitive Memory—VCAP Perceptual speed—VCAP Fluid abilities—VCAP, Task Switching, Spatial Working Memory Vocabulary—VCAP	= = N to C1 = to C2 =

*C* comparator, *NR* not reported, *Cog* cognition, *Y* yes, *N* No, =  equal effect between intervention and comparator groups, *MDBF* Multi Dimensional Mood Questionnaire, *MMSE* Mini-Mental State Exam, *HADS* Hospital Anxiety and Depression Scale, *QoL* Quality of Life, *PANDA* Parkinson Neuropsychometric Dementia Assessment, *ABC* Activities Balance-Specific Confidence Scale, *RBANS* Repeatable Battery for the Assessment of Neuropsychological Status, *PDQ39* Parkinson’s Disease Questionnaire, *VCAP* Virginia Cognitive Aging Project Task Battery, *WMS-III* Wechsler Memory Scale third edition, ? implied, *T1* first follow-up, *T2* second follow-up

*Significant sex differences

**Groups significantly different at baseline; ^a^weight training session duration NR

Measures of adherence included retention of participants in each intervention, rate of attendance, and maintenance of intensity of the intervention. Twelve of the 27 included studies reported greater retention in the dance intervention, and 7 studies reported equal retention. Five reported greater retention in the physical activity comparator group. Three studies did not report the rate of retention in the dance or exercise interventions. The lowest adherence to an intervention was 33% [70]. Only six studies reported attendance at the interventions [62, 65, 78, 83–85], with one study adding detail that attendance was recorded within the first ten minutes of class [84]. Two studies reported on attendance requirements to meet inclusion in statistical analyses [65, 83]. Only two studies accounted for intensity of the interventions as an element of adherence, with one only reporting the intensity for the weight training comparator group [76] and the other study attempting to mimic the aerobic intensity of the dance intervention for the active control group [64].

### Meta-analysis of Psychological Outcomes

**Fig. 2:**
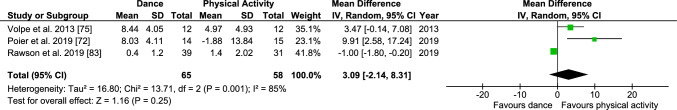
Mean difference in effect of dance compared with physical interventions on PDQ-39. *CI* confidence interval, *SE* standard error

**Fig. 3:**
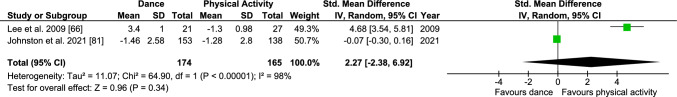
Standardised mean difference in effect of dance compared with physical interventions on anxiety. *CI* confidence interval, *SE* standard error

**Fig. 4:**
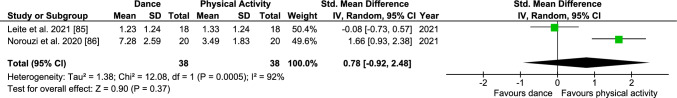
Standardised mean difference in effect of dance compared with physical interventions on Beck Depression Inventory score. *CI* confidence interval, *SE* standard error

The 39-item Parkinson’s Disease Questionnaire (PDQ-39) assesses the frequency with which people with the condition experienced difficulty across eight domains of daily life and its impact on their quality of life. While some items clearly assess mental health, others assess physical health. Based on the three independent studies utilising PDQ-39, meta-analysis did not identify a significant pooled MD estimate (PDQ-39 3.09; 95% CI − 2.13 to 8.30; *Z* = 1.16; *p* = 0.25), suggesting dance interventions did not have a greater effect relative to exercise interventions on how often people with Parkinson’s disease experience difficulties during daily living [72, 75, 83] (Fig. 2). Based on the two independent studies investigating anxiety using a variety of tools, meta-analysis did not identify a significant pooled SMD estimate (anxiety 2.26; 95% CI − 2.37 to 6.90; *Z* = 0.96; *p* = 0.34), suggesting dance interventions did not have a greater effect on anxiety compared to exercise interventions [66, 81] (Fig. 3). The Beck Depression Inventory (BDI) is a 21-item questionnaire that assesses the symptoms associated with depression. Two studies could not be included in the meta-analysis due to insufficient data reported to calculate SMD [70, 80]. Based on the two independent studies investigating depression using the BDI and BDI-II, meta-analysis did not identify a significant pooled SMD estimate (BDI 0.78; 95% CI − 0.92 to 2.48; *Z* = 0.90; *p* = 0.37), suggesting dance interventions did not have a greater effect on depressive symptoms compared with exercise interventions [85, 86] (Fig. 4).

It was not possible to perform meta-analysis on the three studies that reported on quality of life using the 12-item Short Form Health Survey (SF-12) [66], the 36-item Short Form Survey (SF-36) [69], the Life Satisfaction Index (LSI) [69] and the Brief Multidimension Life Satisfaction Scale (BMLSS) [72]. Each of these four outcome measures is composed of varying domains/subscales that measure different constructs.

### Psychological and Cognitive Outcomes in Children (16 Years and Under)

Three studies investigated these outcomes in children. One study [66] examined the effects of Turo (qi gong dance) on negative psychological symptoms such as somatisation (i.e. distress arising from perceptions of bodily dysfunction) and hostility (i.e. thoughts, feelings and actions that are characteristic of the negative affect state of anger) in children under 16 years of age. The Turo group demonstrated significant time × group interactions in the psychological symptoms of somatisation and hostility compared with the control group, who performed Turo movements without the concept of meridian Qi flow. There were no significant differences in other sections of the questionnaire. Two studies found the effect of jazz dance [71] and dance-based physical education classes [82] was equivalent to standard physical education classes in primary school children for cognitive outcome measures.

### Psychological and Cognitive Outcomes in Adults (17–54 Years)

Ten studies [62, 67, 70, 73, 76, 79, 81, 85–87] investigated psychological and cognitive health benefits of dance compared with other forms of physical activity in adults aged 17–54 years. A majority of psychological and cognitive outcomes demonstrated equivalent changes or equivalent lack of changes as a result of the dance and exercise comparator interventions. However, Zumba demonstrated greater improvement in working memory compared with treadmill exercise (*g* = 0.34) [86].

Barene et al. found that soccer improved the self-efficacy and emotional wellbeing of hospital employees after 12 weeks compared with Zumba; however, the improvements were equivalent by the end of the 40-week intervention [62]. Soccer or volleyball also improved depressive symptoms compared with aerobic dance in tertiary students [81]. Bass and colleagues [76] found that weight training was more effective at reducing stress than aerobic dance; however, there were significant between-group sex differences noted and the session duration for the weight training intervention was not reported.

Modern dance demonstrated greater improvements in psychosocial adjustments (*g* = − 9.12) and in cognitive function (*g* = − 9.12) compared with kinesiotherapy in a population of young adults with cerebral palsy [74]. There was no significant difference in cognitive performance (reciting subtractions) of older adults between 1950 to 1970s style dance classes and a combination of strength and endurance training [64]. Both dance and brisk walking improved response time in the flanker test, which examines interference control, with no significant difference between the two groups of elderly females [77].

### Psychological and Cognitive Outcomes in Older Adults (55 Years and Over)

Fourteen studies [63–65, 67–69, 72, 75, 77, 78, 83, 84, 88, 89] investigated psychological and cognitive health benefits of dance compared with other forms of physical activity in adults aged 55 years or older. Eleven of these 14 studies demonstrated equivalent psychological/cognitive changes, or equivalent lack of change as a result of the dance and exercise comparator interventions [64, 67–69, 75, 77, 78, 83, 84, 88, 89]; three studies showed dance had a superior effect [63, 65, 72]. A meta-analysis was unable to be performed on the four studies that included a working memory outcome measure due to the different constructs assessed by each [90]. The Virginia Cognitive Aging Project Task Battery measures spatial memory [89], the N-Back Task measures accuracy of image or letter recall [86], and a list sorting task, presented both visually and auditorily/verbally, measures sequence stimuli recall [71], while the Digit Span Backwards Task measures working memory capacity by recalling a verbal numerical stimulus [68].

A choreographed dance programme combining various forms of dance styles (salsa, rock, rumba, pop and jive) had greater impact on verbal memory recognition than a physical therapy-based exercise intervention (*g* = 1.24) in an older adult population with mild cognitive impairments [63]. Greek dance resulted in greater improvements in intrinsic motivation in the domains of enjoyment/interest (*g* = 2.96), effort/importance (*g* = 3.49) and perceived competence (*g* = 1.76), for participants with chronic heart failure, when compared with a traditional aerobic and resistance training programme [65]. Two studies reported that exercise programmes incorporating strength, flexibility, endurance and balance training were associated with a greater change in verbal fluency in those with amnestic mild cognitive impairment (*g* = 0.71) [63], and in fluency of task switching and spatial working memory in healthy older adults (effect size unable to be calculated) [89], when compared with dance.

Reporting on health-related quality of life, Dahmen-Zimmer and Jansen reported only on the physical sub-score of the SF-12 [78], finding no effect of the dance intervention for their participants with Parkinson’s disease. Kaltsatou and colleagues reported that for their chronic heart failure participants, the Life Satisfaction Index (a measure of QoL), of the Greek dance group correlated positively with time spent exercising and with change in the mental health subscale of the SF-36; however, the authors did not report whether the QoL of participants in the dance group differed from that of the combined aerobic and resistance exercise group [65]. No significant difference in the BMLSS was reported by Poier and colleagues between the group with Parkinson’s disease who undertook Tango dancing compared with those who did Tai Chi [72].

## Discussion

### Overview of Main Findings

Dance was as effective as other physical activity interventions in improving multiple dimensions of psychological and cognitive outcomes (Table 4). The studies included participants across the lifespan (7–85 years) and healthy to chronically diseased samples (i.e. Parkinson’s disease, heart failure, cerebral palsy, fibromyalgia). The dance interventions encompassed a broad range of genres including theatrical dance, aerobic dance, traditional dance forms and social dance, and were compared with a broad range of physical activities including team sport, martial arts, walking and weight training.

There is preliminary evidence to suggest that dance may be superior to other physical activity interventions for the psychological outcomes of motivation [65], distress (hostility and somatisation) [66], depression [86], emotional wellbeing [72] and cognitive outcomes of verbal memory recognition [63], working memory [86] and social cognition (psychosocial adjustments and cognitive function) [74]. Dance was found to be inferior to other physical activity interventions for the psychological outcomes of motivation stress [76], and self-efficacy [78]. Despite generally having higher participation in dance and exercise [91], only five of the included studies investigated psychological and cognitive function in younger people (mean age below 25 years) [66, 73, 74, 76, 79], warranting further research.

### Differences Between Dance and Exercise Across the Lifespan

Even though we can conclude that dance is equivalent to other forms of physical activity in improving a number of psychological and cognitive domains, we cannot make recommendations for a specific population. The differences found are linked to differences in outcome measures used, genres of dance, dosage of the dance and comparator physical activity interventions, and population type (e.g. diseased/healthy). It may be that dance, like physical activity, has different impacts depending on type of dance and age [92].

Physical activity in general elicits benefits to health and wellbeing across the lifespan. However, much like any skill-based activity, self-efficacy for exercise (e.g. perceived motor competence) is a strong predictor of engagement with physical activity, with perceived motor competence being strongly associated with the amount of physical activity regularly undertaken, starting in early childhood and continuing throughout the entire lifespan [93, 94]. Perceived motor competence may influence the likelihood that an individual attempts a new activity later in life [95], and given that many adults and older adults are physically inactive [96], it is reasonable that many adults will need to take up a new physical activity in order to meet physical activity guidelines. Since foundational motor skills easily transfer between similar activities [94], physical activities like those undertaken as a child may be more likely to be attempted than unfamiliar activities. Therefore, if an individual engaged in dance, aerobics or gymnastics as a child, coming to any form of dance as an adult may feel familiar and ‘easy to master’ compared with other forms of exercise. In this way, dance may be considered an approachable activity to begin as an adult; many cultures have traditional dances that will be familiar from one’s youth, removing the barrier of unfamiliarity when approaching a new (dance) physical activity. However, even if an individual has low motor competence for a given activity, the influence of social acceptability, or motivation generated by engaging in an activity with people of a similar age and sex may overcome feelings of poor motor competence and enable sustained engagement with the new physical activity [95, 97, 98]. Indeed, the aspects of ‘social acceptability’ and ‘fun’ are key features of the very successful Zumba movement leading to participation in regular dance as an accessible form of exercise independent of age, body composition and fitness level.

### Selection of Psychological and Cognitive Outcome Measures

The impact of dance on cognitive health outcomes in the oldest age group suggests that dance has additional cognitive benefits beyond those attributed to exercise in older people. This may be because of the combination of exercise and cognitive challenge for which there is emerging evidence of benefits on cognition compared with exercise only [99]. This may not be unexpected, given the interest in interventions that assist in slowing down impacts of aging on cognitive and physical functioning, and enhance quality of life in older adults. However, it presents an opportunity for future researchers to additionally incorporate the measurement of psychological variables within their studies, to explore a broader perspective of the benefits of dance across both domains and potential relationships between the two.

This systematic review provides preliminary evidence that different dance genres have psychological effects in various domains due to specific elements within the dance intervention. For example, Turo (qi gong dance) dance may have therapeutic effects on psychological and stress-related disorders in healthy, female youth [66]. This is consistent with the literature suggesting that arts interventions improve mental health through creative self-expression [100]. Modern dance classes based on Feldenkrais, Horton, Graham and Laban/Bartenieff concepts assisted in improving psycho-social adjustment in cerebral palsy [74] and incorporated elements of somatic practice and awareness which might be akin to psychological mindfulness techniques requiring body and breath awareness. Further investigation is warranted to understand what aspect of dance genres may facilitate being more comfortable with moving one’s own body and being able to express emotions through the medium of dance.

A predominance of social dance genres being investigated for psychological health effects in older adults, combined with largely positive outcomes for these measures (i.e. QoL [65], internal motivation [65], balance confidence [67], emotional wellbeing [78]), raises questions as to whether it is the dancing itself to which the reported positive psychological effects can be attributed or whether it is the highly social nature of the dance activity, or a combination. In contrast to the genres assessed for older adults, six of the seven studies of participants under 55 years examined the effects of dance genres that could be performed individually in a group setting, i.e. aerobic dance [70, 73, 76], somatic dance styles [74], modern dance [79] and qi gong [66], suggesting that it is in fact the dancing that improves psychological health.

The variety of psychological and cognitive outcome measures included makes conclusions across the lifespan difficult. The outcomes may have been selected to reflect particular genres, but the genres themselves may appeal more to older (e.g. social dance) or younger (e.g. modern dance) dancers. Repeating the studies in a broader age group may not be feasible if the preference is not for that dance style. Some outcomes may not have been considered as relevant in different age groups (e.g. working memory in youth), or simply not explored across different genres.

### Considerations for Intervention Design

Whether the psychological and/or cognitive benefits of dance are maintained in the short, medium or long term following these dance interventions is unknown, as only one paper followed up participants after the completion of the intervention. McKinley and colleagues [67] reported on their outcome (activities-specific balance confidence) 1 month after a 10-week intervention of Argentine Tango versus walking, finding maintenance of improved confidence but no between-group difference. Two studies followed up participants after reducing the frequency of the interventions and found equivalent benefit for the dance and exercise interventions [62, 69].

Both the dance and comparator physical activity interventions varied substantially between studies not only with content but also with the exercise ‘dose’. The session length of the dance interventions and their active comparators across the 27 studies ranged from 30 to 90 min and the frequency from one to four times per week, while the duration varied from 6 weeks to 18 months. This variance in session length, frequency and intervention duration consequently made it difficult to draw direct comparisons with regard to the potential generalisability of the findings for psychological and cognitive benefits. Although the physical activity dose was comparable within studies, we cannot be confident that all the physical activity intensities were comparable between studies.

Finally, of interest is whether the intention of the classes for each dance genre was to focus specifically on providing a positive psychological health outcome. While participation in dance classes can often be highly social and interactive, it is not clear whether any specific modifications were made to the delivery of the classes to facilitate enhanced psychological outcomes for the purposes of the research studies, or if the classes were run authentically without any delivery modifications aiming to elicit improvements in the outcome measures. The modern dance class based on somatic concepts like Feldenkrais, the dance-like practice of martial arts forms qi gong and Kata in Karate and potentially yoga classes with specific sequencing may have confounding benefits due to the mindfulness component of their respective philosophies. Research has found that mindfulness can improve psychological health and wellbeing [101]; however, it is unknown if there is any summative effect of simultaneous physical activity and mindfulness of movement that occurs in dance. For future studies, comprehensive reporting on the class design process would help determine the specific intent for delivery of each dance genre and the potential implementation into the community.

### Considerations for Study Quality

The low to moderate quality of the included studies in this review was primarily due to the lack of blinding and the reporting of equivalent treatment for both groups. Overall, the randomised controlled trials (RCTs) included in this review were of a higher quality than the non-randomised controlled trials. The RCTs offered more reliability in their results as follow-up of participants and use of similar groups at baseline for comparison were clearly reported. Bias was also reduced through randomisation, rather than allowing participants to select which intervention they were assigned to. Confusion was noted as studies used the term ‘follow-up’ when referring to measurements taken immediately ‘post-intervention’ rather than true follow-up measurements taken after a wash-out period subsequent to the completion of the intervention.

Two studies demonstrated the highest quality amongst those included [64, 67]. These two RCTs ensured true randomisation, appropriate baseline and outcomes analysis, and reported on comparison groups with similar characteristics. However, it was assessed that both studies lacked appropriate blinding. It is reasonable that it may be difficult to blind participants and the practitioners delivering the interventions to group allocation. However, it is important to not only blind the assessor measuring the outcome variables to group allocation but to also report this blinding in the manuscript. Reporting blinding will help readers determine the mitigated risk of bias that was conducted and how this may impact the researchers’ interpretation of the results. Reporting the full treatment available to participants in a trial will also have a substantial impact on the results of the study. Understanding if the participants are receiving truly equivalent interventions (in terms of total time face to face with clinicians/instructors, advice regarding physical activity or self-management of their conditions, and medication) will ensure that the outcomes measured are more likely to be associated with the different intervention modality.

The results of this study should be analysed with caution due to the low-to-moderate quality of the included studies. Without high-quality studies, confidence in the effectiveness of dance to improve psychological and cognitive outcomes is lower. Improving the quality of research in this field will enable clinicians to implement dance in an evidence-based manner and have confidence that prescribing dance will improve mental health and wellbeing for participants. Research in the dance for health field would benefit from well-structured study designs that incorporate assessor blinding to reduce the risk of bias, and clear reporting of the interventions and treatment conditions to account for confounders that may affect the study results.

### Implications

The findings of this systematic review have implications for both clinical practice and future research. The benefits of dance interventions extend beyond physical health outcomes such as body composition, blood biomarkers, musculoskeletal health and cardiovascular function, to cognitive and psychological wellbeing. Given the similar, if not superior, benefits of dance on mental health and cognition, clinicians aiming to improve these can confidently support patients to choose either a traditional exercise or dance intervention. Exercise that is perceived to be boring is unlikely to improve cognition with the choice of type of exercise linked to enjoyment [102]. Shared decision-making regarding the type of exercise/physical activity to address mental health and cognitive impairment is pertinent to the general population as well as clinical populations (e.g. Parkinson's and cardiovascular disease, cerebral palsy and generalised musculoskeletal pain) from adolescence to adulthood [103].

Enjoyment in an activity improves adherence and sustainability in physical activity [104, 105]. Our systematic review reveals that, of the 24 studies that reported on study retention, 19 found the dance intervention had equal or better retention than the active control. The assumption here is that the recruited cohorts indeed enjoyed the dance intervention. However, enjoyment ratings of the allocated interventions were not collected, and adherence was poorly and inconsistently recorded or reported. All aspects influencing enjoyment should be considered, such as past experiences and autonomous motivation to exercise, because completion of a research study may not translate to sufficient ‘attendance’ at all offered sessions of physical activity or sustainable adherence [106, 107]. For example, simply collecting data relating to attendance does not guarantee compliance with an effective intensity or mental engagement with the physical activity to achieve the proposed psychological or cognitive benefits. Individuals should be encouraged to undertake whichever physical activity modality they are most likely to enjoy, and therefore truly adhere to, to achieve optimal cognitive and mental health benefits.

We propose some recommendations for future research on the relative benefits of exercise and dance interventions on mental health and cognitive impairments. Researchers could consider the effect of shared decision-making regarding group allocation of participants on firstly their satisfaction with the intervention and secondly on their adherence. Follow-up should continue beyond the short-term effects of the interventions. Are participants more likely to continue undertaking some forms of physical activity beyond the period of the study intervention? Is this persistence with the physical activity correlated with their enjoyment, past experience or autonomous motivation? Standardised, reliable and valid assessment tools need to be used in this research to enable comparison of the effects of the interventions.

Since all interventions included in this review required participants to undertake supervised dance or exercise for a minimum of 6 weeks, we suggest this as a minimum period for mental health and cognitive capacity interventions. Those studies that demonstrated superior cognitive and psychological benefits in favour of dance were undertaken for a period of at least two months, two times per week, with session durations between 40 and 60 min.

While pharmacological therapy continues to be standard treatment for mental health impairments, adverse effects influencing drop-out and drug interactions in populations with multiple co-morbidities are possible [108–110]. The high rate of non-adherence to such medications [111] suggests that trials that compare medication with exercise as an adjunct to medication are warranted. Such research has the potential to improve the societal and economic cost of medication as a stand-alone intervention for mental illness.

### Strengths and Limitations

This review was conducted in a rigorous manner, with a priori specifications for all criteria, and all steps were conducted by two researchers and overseen by a third. While only three variables could undergo meta-analysis with a small number of studies eligible for inclusion and high heterogeneity between those studies, the inclusion of a broad range of dance styles and population groups is both a strength and a limitation of the review. Our findings cannot be extended to unplanned and unchoreographed dance forms. Dance is a broad umbrella term that encompasses a wide variety of styles from highly structured, planned movement sequencing to entirely unplanned, intuitive forms of body movement. To examine the effect of dance on psychological and cognitive parameters we chose to narrow the breadth of these dance forms to a more homogeneous inclusion of structured dance to enable equivocal comparison with current exercise guidelines. Our results indicate that the benefits of structured, planned dance to psychological and cognitive health do not appear to be limited to a single dance style or single population group. However, with regard to clinical populations, as this review found five different clinical populations reported over eight studies, these studies need to be replicated before firm conclusions can be drawn about the relative benefits of dance for psychological health.

Other considerations to bear in mind when drawing conclusions from this review, and areas where future research can be improved, include: the short follow-up period in most of the included studies—in which data collection ceased at the conclusion of the active intervention; the uneven sex mix in the dance and control groups—particularly in non-randomised studies in which males tended not to choose the dance intervention; underrepresentation of young people and specific clinical populations for whom dance may theoretically be of extra benefit to psychological wellbeing, such as those with cancer or other chronic diseases [26, 33, 34, 40–42]; and the generally low quality of study design and reporting. There were significant sex disparities between the dance and active comparator groups in three studies [76, 78, 79] that were not accounted for in the analysis of the data. The conflicting evidence regarding sex differences in cognitive functioning and psychological attributes limits the strength of the recommendations made in these studies, suggesting caution is required when interpreting their results [112, 113].

Readers should also be cautious about generalising the findings of this review when implementing dance for health in the community. When designing dance interventions, inclusion of populations underrepresented in the current literature is warranted. For clinicians prescribing dance for patients, individual preference for physical activity modality and/or dance genre may impact the potential benefits of the programme. Many forms of dance are performed to music; however, music was not a factor that was controlled for in this review and this warrants future investigations to distinguish the effect of dance from that of the accompanying music. The evidence appears promising for dance as a suitable alternative to standard exercise programmes in several population groups and situations; however, this field requires more research to replicate those findings and strengthen the evidence for the effects of dance on psychological and cognitive health.

## Conclusions

Notwithstanding limitations, the findings of this review suggest that dance is equivalent or superior to other physical activity interventions to improve psychological wellbeing and cognitive capacity. Structured dance can be considered an evidence-based alternative for individuals who might prefer it to more traditional forms of exercise. In those 54 years and under, the current literature pool focuses on psychological outcomes, while for those 55 years and over, there is a greater focus on cognitive capacity. The effectiveness of dance interventions is most evident in the domains of self-efficacy, anxiety, depression, motivation and health-related quality of life, particularly in older individuals. While there is considerably less evidence in those 16 years of age and below, dance appears to be superior to other exercise in lessening the impact of somatisation and hostility.

## Declarations

### Availability of data and material

Data available upon request.
